# A New Tool to Study the Binding Behavior of Intrinsically Disordered Proteins

**DOI:** 10.3390/ijms241411785

**Published:** 2023-07-22

**Authors:** Aakriti Upadhyay, Chinwe Ekenna

**Affiliations:** Department of Computer Science, University at Albany, State University of New York, 1400 Washington Avenue, Albany, NY 12222, USA; aupadhyay@albany.edu

**Keywords:** intrinsically disordered proteins, protein–protein interaction, geometric features, binding affinity, rigid-body docking

## Abstract

Understanding the binding behavior and conformational dynamics of intrinsically disordered proteins (IDPs) is crucial for unraveling their regulatory roles in biological processes. However, their lack of stable 3D structures poses challenges for analysis. To address this, we propose an algorithm that explores IDP binding behavior with protein complexes by extracting topological and geometric features from the protein surface model. Our algorithm identifies a geometrically favorable binding pose for the IDP and plans a feasible trajectory to evaluate its transition to the docking position. We focus on IDPs from Homo sapiens and Mus-musculus, investigating their interaction with the Plasmodium falciparum (PF) pathogen associated with malaria-related deaths. We compare our algorithm with HawkDock and HDOCK docking tools for quantitative (computation time) and qualitative (binding affinity) measures. Our results indicated that our method outperformed the compared methods in computation performance and binding affinity in experimental conformations.

## 1. Introduction

Intrinsically disordered proteins (IDPs) are involved in many biological processes, such as cell regulation and signaling, and their malfunction can become linked to severe pathologies [1,2,3]. Understanding the functional roles of IDPs requires studying their interactions with other proteins, which is very challenging and needs tight coupling of experimental and computational methods. In contrast to structured/globular proteins, it is not easy to represent IDPs with a single conformation, and their models require ensembles of conformations representing a distribution of states that the protein adopts in solution. Thus, investigation of IDP interaction with structured/globular proteins is indispensable for understanding many biological mechanisms [4]. In terms of applications, understanding such molecular interactions is essential for drug design in pharmacology or protein engineering in biotechnology.

IDPs do not have distinct, well-defined secondary and tertiary structures because of their remarkable backbone flexibility [5]. When an IDP binds to a macromolecule (usually another protein), the large interfaces become involved, resulting in specific but comparatively weak interactions. IDPs common in genomes and proteomes of living organisms have many occurrences in eukaryote groups. They are prevalent in various human diseases and enriched in cardiovascular disease, diabetes, cancer, and neurodegenerative disease-related proteins [6]. The disordered region can arise spontaneously because millions of copies of proteins get generated during the lifetime of an organism, making humans an easy target for many infectious diseases during host–pathogen interactions [7,8]. Pathogen-like *Plasmodium falciparum* (PF) is a protozoan parasite of humans that inflicts damage to the human immune system and is responsible for most malaria-related deaths [9]. Plasmodium infection in mammals begins with the injection of the sporozoite into the skin of the vertebrate host through the bite of a female Anopheles mosquito. This results in growth and multiplication, first in the liver cells and then in the red blood cells, leading to kidney failure, severe anemia, and many more consequences [10]. We considered host–pathogen interaction between the PF pathogen and human/mice IDPs to study and analyze the binding behavior of IDPs in structure-based molecular interactions.

The intrinsic disorder poses a challenge for both experimental analyses of the conformation and computational modeling due to the lack of stable structure. In spite of the instability, it is critical to understand the biological functionality during protein–protein interactions. Several rigid-body docking techniques have emerged as helpful tools to assess the prediction of possible interacting poses between two protein bio-molecules for global docking [11]. These docking servers sample conformations of the smaller protein bio-molecule around the larger one and use the scoring functions to determine the top docking predictions. ZDOCK [12], RDOCK [13], and pyDock [14] use fast Fourier transform (FFT)-based algorithms; RosettaDock [15] is built on a Monte Carlo (MC)-based multi-scale docking algorithm; and FRODOCK [16] and HDOCK [17] use a knowledge-based approach to predict the translational and rotational orientation of the interacting proteins. Another tool, HawkDock [18], uses the ATTRACT [19] docking algorithm to predict several binding poses and determines the near-native docking using the HawkRank score. However, these tools do not considered the topology and geometry of the protein to analyze the binding site for structure-based molecular docking.

**Figure 1:**
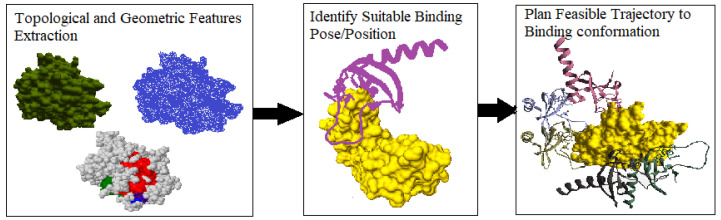
Workflow of our approach.

In this work, we propose a topology-based rigid-body docking algorithm that takes the protein surface models of the globular proteins to predict a binding conformation for the interacting IDPs. Our approach extracts the topological and geometric properties of the protein surface to generate random IDP conformation ensembles around it. It then ranks the conformation ensembles based on the docking score to find the geometrically favorable pose. The algorithm examines the score values to select the geometrically favorable binding position and plans a feasible trajectory from IDP’s initial location to it. Our method can be used as a tool to find the best docking position that geometrically fits the protein surface model when no information other than the individual structures is available. Figure 1 shows an overview of our workflow.

We performed experiments for nine globular proteins interacting with six IDP molecules in the conformation space. We considered the tertiary structure of the globular proteins, ranging between 173 and 1544 residues, as a stationary object and the rigid body of the IDPs as a moving object. Our results showed improved quantitative (i.e., computation time) and qualitative (i.e., binding affinity) analyses with our tool compared to two publicly available tools; i.e., HawkDock and HDOCK. We evaluated the interaction of all IDPs with all proteins over 1250 experiments with values averaged over 10 runs in each case and planned a path to the binding pose with the highest score among the top 10 predicted conformations.

## 2. Results

### 2.1. Experimental Data

**Figure 2:**
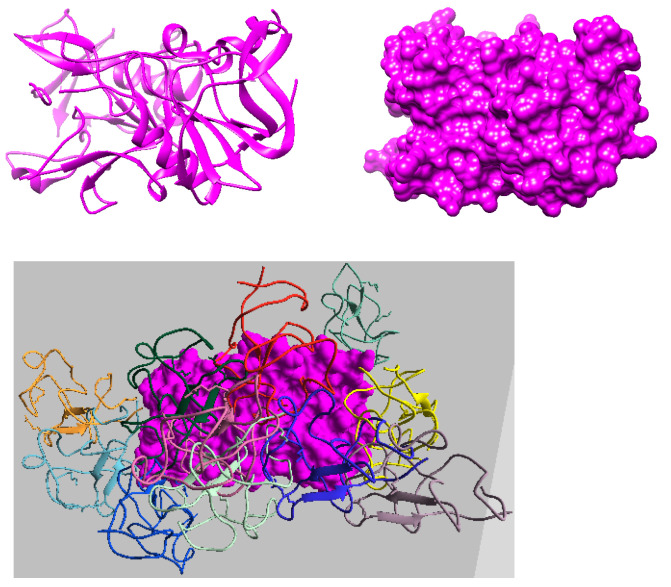
The figure shows the 3SRI protein, its multiscale surface model, and the predicted IDP conformations around detected geometric features (critical points) of the protein surface. The IDP conformations are the top 10 predicted docking positions of 1KRN bio-molecule around the surface model.

We obtained protein data from the Protein Data Bank (PDB) [20,21] and constructed their tertiary structures using CHIMERA [22]. We obtained IDP data from the PDB and AlphaFold Protein Structure Database (AlphaFold DB) [23]. We considered nine proteins and six IDP bio-molecules to study and understand the biological binding mechanism of IDPs using protein surface geometries. The high-dimensional surface models of the proteins represented them as stationary rigid bodies in the conformation space. Figure 2 shows the tertiary-structure representation of the 3SRI protein, its high-dimensional surface model, and the IDP conformation ensembles around it.

**Figure 3:**
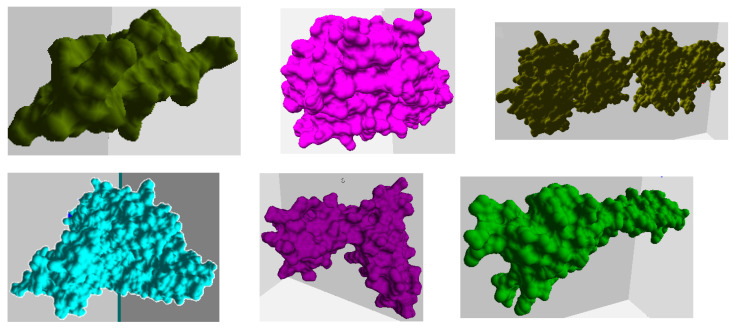
The figure shows the tertiary structure of the PF pathogen proteins in the order 2MU6, 3SRI, 4JUE, 4M1N, 6ZRY, and 7F9K.

**Figure 4:**
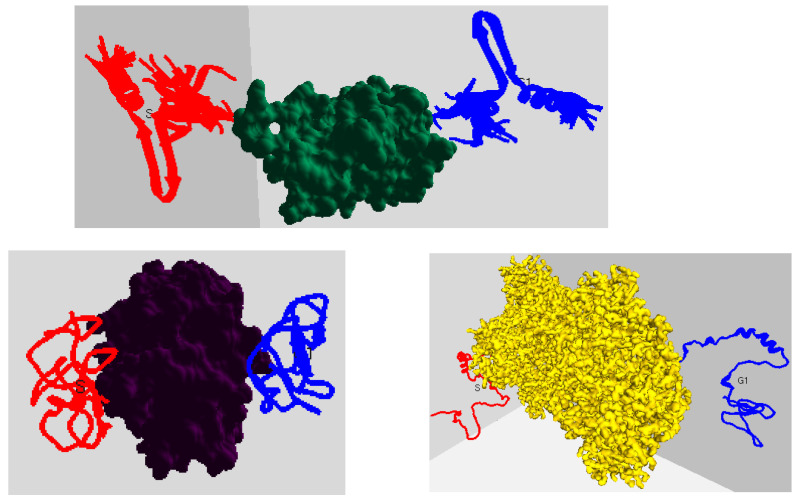
The figure captures a random combination of a globular protein surface model and an IDP from the experimental analysis, given in the sequence as 1SQ6 (2LE3), 1TQX (1KRN), and 3NTJ (AF-I1E4Y1-F1). The IDP names are mentioned in brackets. The red-colored conformation refers to the start position, and the docking position is in blue.

The proteins selected included nine *Plasmodium falciparum* (PF) pathogen proteins (i.e., 1SQ6, 1TQX, 2MU6, 3NTJ, 3SRI, 4JUE, 4M1N, 6ZRY, and 7F9K), as shown in Figure 3 and Figure 4. PF is responsible for most malaria-related deaths and forms part of our ongoing research into identifying feasible protein drug targets. The high mutational capacity, coupled with the changing metabolism of the pathogen, makes the development of malaria drug treatments an evolving problem. In this work, we were interested in studying and analyzing the behavior of PF pathogens in the PPI network. Hence, these proteins were selected as they are the potential targets for malaria infections.

We selected the 1KRN (88 residues), 2LE3 (42 residues), 5EJW (91 residues), and 7KPI (142 residues) proteins as IDPs based on their highly disordered behavior shown in the protein feature view plot available in the PDB database. The other IDP bio-molecules, AF-I1E4Y1-F1 (117 residues) and AF-P59773-F1 (190 residues), from the AlphaFold DB, were of the mus-musculus and homo sapiens species, respectively. The mean per-residue confidence score (pLDDT) for AF-I1E4Y1-F1 is 48, and for AF-P59773-F1, it is 59. The pLDDT measure estimates whether the predicted residue has similar distances to neighboring C-α atoms (within 15 Å) in agreement with the naive structure and scores them between 0 and 100. The score assesses the local model quality of the structure; i.e., a lower score refers to the existence of more disordered regions in a bio-molecule. The selected IDPs were bio-molecules from humans and mice susceptible to malaria.

Figure 3 shows the surface models of six PF pathogen proteins, and Figure 4 shows a random combination of IDPs interacting with the remaining three proteins at their start (red) and goal (blue) positions. We conducted tests on every PF protein complex with all 6 IDPs, resulting in a total of 54 protein–protein complexes. The details for these interactions can be found in Table 1.

### 2.2. Experimental Analysis

We performed experiments on a Dell Alienware Aurora desktop machine running the Ubuntu 20.4 LTS operating system and developed algorithms in C++ language using the PMPL library [24]. We evaluated performance using quantitative and qualitative measures for all IDPs with each PF protein for geometric feature extraction, path planning to dock position, and binding affinity. Overall we executed 1250 experiments and averaged the result values over 10 runs. We compared our method’s performance with two baseline methods; i.e., HawkDock [18] and HDOCK [17].

#### 2.2.1. Quantitative Analysis

Extracting Geometric Features of the Protein Surface: Recall that our method constructs a surface mesh (or simplicial complex) around the considered protein surface models to abstract the topological and geometric information. During the execution, it randomly samples the IDP conformations around the protein surface model, thus constructing a manifold mesh representation to capture the topology of the protein’s surface. These topological features aid in identifying the geometric properties of the protein surface (i.e., minima or maxima) for better approximations, as shown in Figure 1. Next, our method uses geometric information to find possible binding conformations around the protein surface and applies the scoring function from Equation (2) to obtain the top 10 geometrically fitting docking conformations for an IDP. Figure 2 shows the top 10 predicted association conformations of the 1KRN IDP around the 3SRI protein surface model. We observed that the feature extraction process was independent of the globular protein’s size and had minimal effect on the performance of our algorithm, making it suitable for macro-molecules, as discussed next.

**Figure 5:**
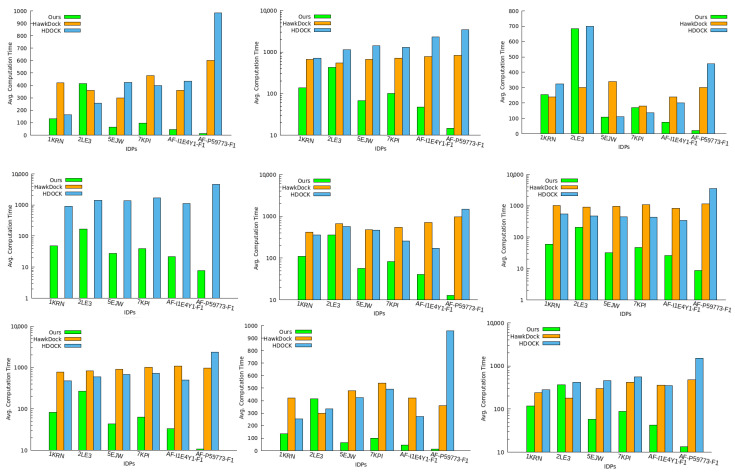
The plots show the total computation time taken (in seconds) by all three methods to predict the top 10 IDP docking conformation ensembles around the protein surface model. The results in the plots are shown for PF protein conformation space in the sequence as 1SQ6, 1TQX, 2MU6, 3NTJ, 3SRI, 4JUE, 4M1N, 6ZRY, and 7F9K.

Computational Time: We analyzed the computation time (in seconds) required for feature extraction and prediction of geometrically favorable docking conformations in these IDP–protein interactions. This study included all IDPs in nine PF pathogen protein conformation spaces. The feature extraction time measures the duration of the extraction of the topological and geometric features from the globular protein surface, while the ranking time finds the top 10 conformations. To assess our algorithm’s performance efficiency, we compared our total time to output the top 10 docking conformations with HawkDock and HDOCK, as depicted in Figure 5.

Our method demonstrated faster prediction of IDP binding conformations compared to HawkDock and HDOCK in all PF pathogen protein conformation spaces except for 2LE3. The smaller size of the 2LE3 IDP led to a longer conformation sampling time, necessary for accurate feature capture of large- or complex-sized proteins. Proteins like 1SQ6, 6ZRY, and 7F9K have tetrahedral polygonal shapes rather than spherical surface structures, thus allowing the sampling of conformation ensembles in various fitting positions. As a result, it took extra time in the case of the 2LE3 IDP within the conformation space of these proteins. However, this difference did not affect our method’s performance significantly and resulted in lower time overhead compared to the baseline methods. Figure 5 highlights that our method outperformed the baseline methods in most protein conformation spaces, despite the minimal time overhead.

In particular, HawkDock failed to find docking conformations for the 3NTJ protein due to its limitation to proteins with fewer than 1000 amino acids.

We found that, using the geometric information for the protein surface, it was still possible to predict multiple structural arrangements of IDPs around the proteins to find the closest interacting binding pose between two bio-molecules without declining computation performance. Thus, we can conclude that the amount of data assessed by our method does not impact its surface approximation, and it still provides a quantitatively good performance.

#### 2.2.2. Qualitative Analysis

Selecting the Suitable Binding Conformation: As initially mentioned, our method predicted the top 10 docking conformations for an IDP across all PF pathogen protein conformation spaces. This process was iterated over 10 times, and for each iteration, we recorded the top 10 conformations to assess the likelihood of obtaining the same conformation from 10 random iterations. The recorded outputs were then further analyzed to identify the IDP conformation with the highest frequency as the most suitable docking position among the 10 experimental runs. This selected conformation was subsequently utilized as input for path planning. In Figure 4, examples of the best binding poses (goal positions) for three IDPs are depicted in blue. To validate the quality of the chosen binding pose for protein–protein interactions, we examined the binding affinity before proceeding with path planning, as elaborated in the subsequent discussion.

Binding Affinity Measure: We compared the binding affinity of our IDP binding conformation with the binding affinity computed for the IDP conformations predicted by the HawkDock and HDOCK methods across all PF pathogen proteins. The molar Gibbs free energy ΔG was used to assess the relevance of the binding pose. Gibbs free energy is a thermodynamic potential that quantifies the maximum reversible work capacity of a thermodynamic system under constant temperature and pressure (isothermal, isobaric) conditions [25]. Protein binding occurs when the change in Gibbs free energy ΔG is negative, indicating equilibrium at constant pressure and temperature.

**Figure 6:**
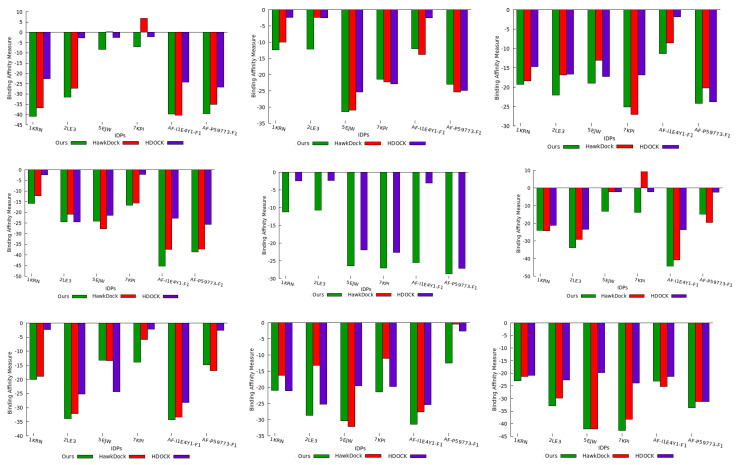
The plot shows the binding affinity measures for the topmost IDP docking conformations predicted by the three methods. The results in the plots are shown for PF protein conformation space in the sequence as 1SQ6, 1TQX, 2MU6, 3NTJ, 3SRI, 4JUE, 4M1N, 6ZRY, and 7F9K.

We utilized the molar Gibbs free energy ΔG to calculate the binding affinity of the top-ranked IDP conformation ensemble predicted by all three methods. Figure 6 illustrates the binding affinity measures of our predicted IDP binding pose for each protein compared to the binding affinity measures obtained for the IDP conformations predicted by the HawkDock and HDOCK methods. HawkDock exhibited a positive binding affinity for the 7KPI IDP conformation when interacting with the 1SQ6 and 4JUE proteins. In contrast, our algorithm consistently predicted IDP conformations with negative binding affinities for all IDPs interacting with PF pathogen protein complexes. This evidence indicated that a stronger association was displayed by our geometrically favorable docking positions and greater consistency was achieved in identifying favorable binding conformations through our method.

As mentioned previously, HawkDock failed to predict the docking conformation for the 3NTJ protein, resulting in the absence of a binding affinity measure for this case.

Based on the observations in Figure 6, we consistently found that our predicted docking conformations exhibited negative binding affinity for all IDPs, surpassing the binding affinity of the IDP conformation ensemble generated by the baseline methods. Additionally, we deduced that our method performed well even for macro-molecule proteins, such as 3NTJ, surpassing HDOCK and not being limited to small bio-molecules. Overall, our experimental conformations demonstrated better binding affinity in 95% of the compared cases, highlighting the significance of utilizing protein surface model features in generating conformations with favorable binding affinity outcomes. Consequently, we can conclude that the quality of our binding conformations competes favorably with the binding conformations predicted by existing approaches utilizing coarse-grained force-field docking (HawkDock) and knowledge-based template-free docking (HDOCK).

Affinity Comparison to Known IDPs: We analyzed the binding affinity of protein–protein complexes specifically by focusing on folding-upon-binding [26]. To validate our method-predicted docking conformation, we compared our results with the work undertaken in [27,28,29], which studied the interaction mechanism of IDPs in terms of structure, dynamics, affinity, and kinetics. To examine our results, we considered the same protein complex compounds as those studied in the aforementioned work; i.e., 4HTP, 3W1G, 3ALO, and 1SB0. Table 2 displays the binding affinity of the known and our predicted docking conformations for these protein complexes. Our findings revealed that our geometrically fitting binding conformation exhibited binding affinities that were highly similar to the known binding affinities of these protein complexes.

#### 2.2.3. Path Planning to Geometrically Favorable Binding Position

**Figure 7:**
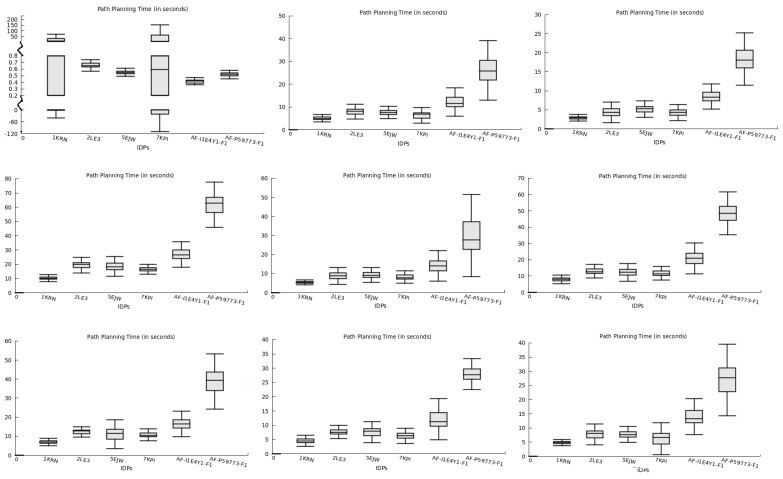
The total time taken (in seconds) to plan a path for all IDPs in each protein’s conformation space. The results in the plots are shown for PF protein conformation space in the sequence as 1SQ6, 1TQX, 2MU6, 3NTJ, 3SRI, 4JUE, 4M1N, 6ZRY, and 7F9K.

In addition to predicting binding conformations for rigid-body docking, our method also included feasible trajectory planning toward the selected finalized binding pose during re-scoring. We assessed the total time required for path planning to the predicted binding pose for all IDPs in the nine globular protein conformation spaces, as presented in Figure 7. The path planning time represents the duration required for an IDP to transition from its initial conformation to the binding conformation while moving closer to the protein surface.

Figure 7 illustrates the distribution of path planning times for all IDPs across different protein conformation spaces. The y-axis represents the averaged path planning time over 10 runs, while the x-axis represents the IDPs interacting with the respective proteins. The plot showcases the variability in path planning time, depicting the duration needed to move IDPs from their initial positions to the docking positions around the protein surface. In several protein conformation spaces, the differences between the minimum and maximum planning times were small or negligible for IDPs exhibiting lower deviations, indicating that the planner consistently found a similar route for the majority of times out of the 10 runs. However, the 1KRN and 7KPI IDPs in the 1SQ6 protein’s conformation space required longer time spans. This behavior can be attributed to the broader structure of these IDPs, affecting their movement near the 1SQ6 protein surface and resulting in varying time values. Among all the studied IDPs, AF-P59773-F1 demonstrated a vast structure and the most disordered regions, making it challenging to plan its path while considering its structural transformations. Thus, we deduced from Figure 7 that the path planning period for the AF-P59773-F1 IDP was generally longer than for other IDPs in most protein conformation spaces.

The unpredictable behavior of IDPs around the studied proteins enabled us to analyze the feasibility of their interaction with specific proteins, particularly how easily they aligned around a protein structure for association. Path planning time provides insights into the locomotion of IDPs around proteins as they search for the most suitable binding pose for rigid-body docking. When used in conjunction with other tools to examine the conformational flexibility of IDPs during their motion around proteins, it can simplify flexible docking tasks by focusing computational methods solely on the dynamic structure of IDP conformations as they traverse the planned trajectory, facilitating future biological studies.

**Figure 8:**
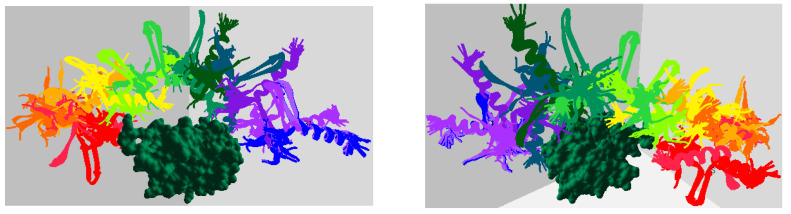
The figures display paths planned for the 2LE3 IDP around the 1SQ6 protein surface model using the geometrically favorable conformation ensembles. The start conformation is in red, and the binding goal position is in dark blue. The pictures, from left to right, show the front and top view of the path planned during experimental analysis.

Figure 8 displays screenshots of the planned path for the 2LE3 IDP around the 1SQ6 protein surface, depicting the motion of the IDP biomolecule from its initial position to the experimentally predicted binding pose conformation. Different view angles illustrate the IDP’s movement around the protein surface, and the intermediate conformations represent the IDP conformations generated during feature extraction, serving as waypoints. These intermediate conformations between the starting and goal positions demonstrate the structural transformations of the 2LE3 IDP as it moves in the vicinity of the 1SQ6 protein surface. Similar movements and structural arrangements occurred for the remaining IDPs across different protein conformation spaces.

We conclude that our approach successfully captures the geometric features of the protein surfaces and plans a path for IDP bio-molecules to the geometrically favorable binding pose, showing a higher affinity compared to affinity measures demonstrated by baseline methods. Thus, the work showed the significance of our approach for further biological studies.

## 3. Discussion

An important area of study includes understanding how a protein binds to another protein’s active site and what conformational changes both molecules undergo during docking to the active site or exit from it. Such information allows for predicting the possibility of an association between protein–protein pairs, the strength of this association, and the protein activity level. Protein function evaluation is a challenging task approached by various sequence-based and structural-based methods [30]. However, the fact that the function of a protein is intrinsically related to its 3D conformation (more than to its primary sequence) motivates the use of structure in predicting protein function [31,32]. During protein–protein interactions, the geometrical structure of the underlying topological manifold plays crucial roles that affect specific biologically related functions, such as driving the cellular immune response [33]. To this end, various developed computational approaches predict the 3D conformation for molecular docking [34,35,36,37], where the bio-molecules bind to the protein regions with potential coherence in the matching (concave) curvatures. The authors of [38] presented an AutoDock-based incremental protocol (DINC) that addresses the limitations of AutoDock’s standard protocol by enabling improved docking of large bio-molecules. DINC performs docking using AutoDock incrementally instead of in one single step by dividing the docking problem into smaller sub-problems.

Another interesting docking conformation prediction tool in [18] integrates the rigid-body docking protocol of the ATTRACT [19] docking algorithm to predict several binding poses and determines the near-native docking using the HawkRank score. Similarly, HDOCK [17] is a hybrid docking algorithm that combines template-based modeling and template-free docking. The method overcomes misleading templates by switching to a template-free docking protocol and calculates the docking energy score using a knowledge-based iterative scoring function. However, these docking servers are limited to the number of residues or the size of the receptors, which results in the failure or degradation of their performance. Our method overcomes this limitation by focusing on the features of the protein surface model independent of its size. Research in [11,39,40] reviewed rigid-body docking methods and experimentally showed that rigid-body docking provides better accuracy than flexible docking. In this work, we perform rigid-body docking to evaluate the binding behavior of IDPs in interaction with globular proteins.

Many studies have used a graph representation of a protein, indicative of its geometry and topology, to predict protein function [41]. The topology of protein bio-molecules has been shown to be surprisingly effective in simplifying bio-molecular structural complexity, attracting attention to gaining a better understanding of bio-molecular behavior during protein–protein interactions. The authors of [42] proposed a set of topological methods to examine possible biases introduced in protein–protein interaction network data. Menglun et al. in [43] presented a topology-based network tree to predict PPI using convolutional neural networks (CNNs). They characterized PPIs using an element- and site-specific persistent homology. Likewise, the authors of [44] introduced an ensemble learning approach for PPI prediction that integrated multiple learning algorithms and different protein-pair representations. Unlike the discussed strategies, we utilized the topological information for the protein surface to extract the geometric features that help predict the IDP conformation ensembles. Our method benefits from using topological data analysis tools rather than deep learning methods and overcomes the supervised learning time overhead for precise feature extraction.

### 3.1. Studied Biological Mechanisms of IDPs

Studying the conformation of highly dynamic IDPs is a challenge in structural biology [45]. Nuclear magnetic resonance (NMR), often used in the study of IDPs [46], is a versatile spectroscopy method for studying proteins that, importantly, do not require crystallization. However, NMR spectral data from IDP ensembles have provided conformational constraints. NMR-constrained molecular dynamics (MD) [47] simulations need multiple copies of the protein (known as replicate exchange MD) to generate possible structural models, which fails to ensure the validity of the result, regardless of the method used to sample the conformations using NMR data.

The authors of [48] used NMR to characterize the structure and dynamics of IDPs in various functional states and environments. They described the NMR parameters of the structural ensemble to quantify the conformational propensities of IDPs and the challenges associated with obtaining structural models of dynamic protein–protein complexes involving IDPs. Another survey [49] summarized the recent developments in computational IDP drug design strategies and analyzed the typical properties of reported IDP-binding compounds (iIDPs) as potential drug targets. Researchers have used the combination of molecular dynamics simulations and circuit topology (CT) to analyze the biological behavior of a human androgen receptor with a large N-terminal domain (AR-NTD) [50]. The method involved constructing the circuit topology of a potentially charged bio-molecule to analyze the fluctuations in the chain using the root-mean-square fluctuation (RMSF) and root-mean-square deviation (RMSD) metrics. Although the interaction of IDPs with other bio-molecules is a critical problem that needs a good understanding of IDPs’ functionality for drug design, there has been little effort devoted to investigating the behavior of IDPs using the surface properties of the binding protein. As a result, we took the first step to evaluate the binding behavior of IDPs through our algorithm using the topological and geometric properties of the bio-molecules.

### 3.2. Sampling-Based Motion Planners (SBMPs)

A particular domain of molecular modeling relates to the prediction of the binding structure of protein–protein complexes; this problem is usually addressed with computational methods. The method is required to accurately predict the 3D conformation of the bio-molecule upon binding to the target receptor. A new research area has tried applying robotics-based motion planning techniques to this problem [51,52,53,54], randomly sampling alternative conformations, in consideration of the position and orientation of the bio-molecule inside the receptor’s binding cleft, planning a feasible path to the binding conformation. The space under which the *degrees of freedom* (i.e., the number of parameters, like residues or C-α atoms, needed to describe the pose) of a bio-molecule is explored is called the conformation space and the regions free of all internal and external constraints are called the $C_{free}$ space in the conformation space.

Vojtĕch et al. in [55] used the Rapidly Exploring Random Tree (RRT) algorithm [56] to explore the void space in each frame of the protein dynamics to reconstruct a dynamic tunnel by back-tracking the tree towards the active site. The tunnel paths from an inner protein active site to its surface provide insights into important protein properties (e.g., their stability or activity) in the interaction network. The authors of [53] provided a proof-of-concept for mimicking ligand flexibility in rigid body molecular docking that can run efficiently in commodity hardware. The method simulates user search performance with a path optimization algorithm for interactive molecular docking. The authors of [57] presented a hybrid algorithm that combines Monte Carlo sampling and RRT* to explore conformational pathways for large-scale motions. The method improves upon the authors’ previous work to produce optimized conformational routes through accurate and efficient searches of the conformational space. Recent work in [58] proposed a parallel implementation of a multi-tree variant of the Transition-based Rapidly Exploring Random Tree (TRRT) that globally explores the conformation space for the IDPs. The method performs a randomized exploration of the conformation space to find probable transition paths between stable states of a molecule using the potential energy cost map. Instead, we utilized the topological and geometric properties of the protein structure to generate the IDP conformation ensembles around it.

In this work, taking inspiration from our prior work, we present a new bio-topology algorithm that randomly explores the rotational and translational *degrees of freedom* of IDPs without exploring their conformational flexibility for rigid docking. The approach utilizes the topological and geometric properties of the protein surface to identify the geometrically suitable structure arrangement of an IDP around a protein receptor and finally plans a feasible path to it.

## 4. Materials and Methods

### 4.1. Mathematical Definitions

We discuss some of the mathematical concepts used in our algorithm to extract the topological and geometric features of the protein surface.

**Definition 1.**  *(Abstract simplicial complex). An abstract simplicial complex K (i.e., a collection of sets closed under the subset operation) is a generalization of a graph that is useful in representing higher-than-pairwise connectivity relationships.*

The elements of the set are called vertices, and the set itself is a simplex. The vertices refer to the IDP’s conformation in the conformation space.

**Definition 2.**  *(Vietoris–Rips complex). Given a set S of points in Euclidean space E, the Vietoris–Rips complex R(S) is the abstract simplicial complex whose k-simplices are the subsets of k+1 points in S with a diameter that is at most ε*.

In this work, protein surface models were considered static objects. *S* defines the group of all IDP conformations in the simplicial complex R(S). These conformations are generated at a radial distance 2ϱ away from the surface to avoid collisions, such that $S\subseteq C_{free}$. We take ϱ as the diameter of the circumscribed circle of the IDP bio-molecule. Considering the above parameters, we define the discrete Morse function as follows.

**Definition 3.**  *Let D be the Euclidean distance function that measures the distance between the point $x\in C_{free}$ and the nearest point y on the protein surface P; that is, $D\left(x\right)=\min_{y\in P}∥x-y∥$*.

**Definition 4.**  *Let Γ(y,ϱ) be a density function where ϱ> 0 and y be the point on the protein surface. The function* Γ *counts all neighbors close to y in S within the distance ϱ*.

**Definition 5.**  *Let f be a discrete Morse function on R(S) restricted to the vertices of the Vietoris–Rips complex. We formally define f at any point in the conformation space by*

(1) f(x)=D(x)·Γ(y,ϱ).

*Please refer to [59] for our expanded definitions and theorems.*

**Definition 6.**  *(Critical points). The set of critical points is defined as the set of non-degenerate points on the surface of the protein when the given discrete Morse function f reaches its extreme values; i.e., local minima or maxima.*

**Definition 7.**  *(Feasible critical points). This set is defined as all possible IDP conformations in S at a radial distance of ϱ from a critical point on the protein surface. In other words, it is the union of intersections of vertices in S within the metric balls of radius ϱ centered at some critical point.*

Overall, our method first generates a simplicial complex R(S) that captures the topological structure of the globular protein surface; i.e., vertices, edges, and triangles. Then, it applies the discrete Morse function on the same simplicial complex to extract the critical point information for the surface and identify the feasible critical points (i.e., IDP conformation ensembles) close to the surface. The discrete setting of Morse theory avoids the overhead of differential geometry, thus reducing the computation complexity for high-dimensional structures. The upcoming section discusses the algorithmic details of our method.

### 4.2. Finding a Suitable Docking Conformation

Algorithm 1 constructs a simplicial complex around the protein surface by sampling and connecting IDP conformations using the method *ConstructComplex*. Upon satisfying the sampling condition from [60], the algorithm performs a topological collapse to remove redundant topological information (i.e., vertices and edges) and provides a skeleton of the simplicial complex around the protein surface in line three; i.e., a surface mesh. Recall that we refer to vertices as the IDP conformations, and the edges are the lines that connect the to/from movements of the IDP between two conformations. The method applies the discrete Morse function *f* from [59] to this simplicial complex to identify the local maxima (protrusion) and minima (cavity) curvatures of the protein surface (i.e., critical points) in line four. The identified critical points are the highest and the lowest peak points on the surface at which function *f* reaches its extremum.
**Algorithm 1** Sampling and planning path to binding pose**Input:** *P*: Protein surface model, *R*: A planned pathway to the binding site, *s*: initial IDP conformation, *H*: set of closest IDP conformations around the protein surface, *g*: best binding pose.1:Let R←{ϕ}.2:S←ConstructComplex(P); ◃ Refer to Definition 23:TopologicalCollapse(S); ◃ Refer to [60]4:C←IdentifyCriticalPoints(S); ◃ Refer to Definitions 5 and 65:F←GetFeasiblePoints(S,C); ◃ Refer to Definition 76:**for all** x ∈ F **do**7:     **for all** c∈C **do**8:          **if** x closest to c **then**9:               $H\left[x\right]=d_{pose}\left(x,c\right)$◃ Refer to Equation (2)10:          **end if**11:     **end for**12:**end for**13:$g=\forall_{x\in H}min\left(H\right)$14:R=PlanPath(s,g)◃ Refer to [61]15:**return** {S⋂F,R}

The algorithm then extracts the feasible critical points at radial distance ϱ from the identified critical points of the protein surface in line five. These feasible critical points are the conformations in close proximity to the protein surface and are part of the simplicial complex R(S) (refer to Definition 7). Next, we consider the closest conformations as the set of predicted conformations for an IDP and use them to evaluate and determine the ranks of the conformations with Equation (2). From the predicted conformations, a geometrically favorable binding position for the IDP is selected such that the conformation is closest to the protein surface curvature in lines 9–14. We use the Hausdorff distance to measure the distance between the protein surface (P) and the IDP conformation (I) to find the geometrically suitable docking position, as discussed below.

(2) $$d_{pose}\left(P,I\right)=\max\left\{\underset{p\in P}{\sup}\underset{i\in I}{\inf}d\left(p,i\right),\underset{i\in I}{\sup}\underset{p\in P}{\inf}d\left(p,i\right)\right\}.$$

The method takes the conformation with the minimum Hausdorff distance as the final docking position from all the possible generated conformations. Finally, a path is planned for the IDP from the start conformation to the binding pose conformation, taking the other predicted IDP conformations as waypoints (line 14). The process of selecting a binding pose happens internally, with our method ranking and automatically choosing the binding conformation to plan the path during the interaction of protein–protein complexes. As a result, our algorithm outputs an extracted geometric information map consisting of critical points, feasible critical points (predicted IDP conformations), and a pathway from the start conformation to the binding pose conformation.

## 5. Conclusions

The paper presented a framework that utilizes topological and geometric information from the structured protein surface to investigate the binding behavior of IDPs. The study assessed the performance efficiency and quality of the predicted experimental IDP conformations, comparing them to state-of-the-art methods. Additionally, it reported the path planning time required to determine a transition path to the docking position.

The experimental results demonstrate that our method successfully predicts geometrically suitable binding poses for IDPs around protein surface models, specifically for rigid docking. Moreover, our approach outperformed the compared methods in computational performance and the predicted conformation quality. This research serves as an initial step towards further analyzing IDPs and their interactions with other biomolecules, leveraging geometric and topological representations of these entities. The future enhancement will incorporate scoring functions and binding affinity measures in our model by integrating it with computational methods designed to estimate binding affinities, with the benefit of determining the final association site for dynamically unstable IDPs more effectively. We plan to apply this idea to our future work and provide a prototype accessible to the research community. By achieving a geometrically suitable conformation with the lowest score and a high binding affinity, our approach presents the potential for advancing the development of structure-based vaccine design processes.

## Figures and Tables

**Table 1:** Protein interaction complexes.

PF Proteins	Interacting IDPs
1SQ6	1KRN, 2LE3, 5EJW, 7KPI, AF-I1E4Y1-F1, AF-P59773-F1
1TQX	1KRN, 2LE3, 5EJW, 7KPI, AF-I1E4Y1-F1, AF-P59773-F1
2MU6	1KRN, 2LE3, 5EJW, 7KPI, AF-I1E4Y1-F1, AF-P59773-F1
3NTJ	1KRN, 2LE3, 5EJW, 7KPI, AF-I1E4Y1-F1, AF-P59773-F1
3SRI	1KRN, 2LE3, 5EJW, 7KPI, AF-I1E4Y1-F1, AF-P59773-F1
4JUE	1KRN, 2LE3, 5EJW, 7KPI, AF-I1E4Y1-F1, AF-P59773-F1
4M1N	1KRN, 2LE3, 5EJW, 7KPI, AF-I1E4Y1-F1, AF-P59773-F1
6ZRY	1KRN, 2LE3, 5EJW, 7KPI, AF-I1E4Y1-F1, AF-P59773-F1
7F9K	1KRN, 2LE3, 5EJW, 7KPI, AF-I1E4Y1-F1, AF-P59773-F1

**Table 2:** Binding affinity comparison for known IDPs.

Protein Complex	IDP	PDB Compound	$\delta G_{\mathrm{Known}}$	$\delta G_{\mathrm{pred}}$
$Artemis^{457–502}$	DNA ligase IV	4HTP	−7.7	−8.1
$Artemis^{593–621}$	DNA ligase IV	3W1G	−6.9	−5.9
p38 peptide	MKK4	3ALO	−3.7	−5.11
KIX domain of CBP	c-Myb	1SB0	−7.3	−7.5

## Data Availability

The data presented in this study are openly available in Protein Data Bank (https://www.rcsb.org/ (accessed on 12 April 2023)) and AlphaFold Protein Structure Database (https://alphafold.ebi.ac.uk/) accessed on 12 April 2023.

## Abbreviations

The following abbreviations are used in this manuscript:

IDPs	intrinsically disordered proteins
PPI	protein–protein interaction
PF	Plasmodium falciparum
MKK4	dual-specificity mitogen-activated protein kinase kinase 4
CBP	CREB-binding protein
CREB	cAMP-response element binding
